# Unveiling bioactive compounds in kola nut seeds: GC-MS identification and computational analysis for anticancer potential

**DOI:** 10.3389/fnut.2025.1706709

**Published:** 2026-01-09

**Authors:** Azzah Alshehri, Saleh Bahaffi, Mohd Rehan, Mohd Suhail, Ahmed I. Al-Asmari, Diaa T. A. Youssef, Alaa Khedr, Torki Zughaibi

**Affiliations:** 1 Department of Chemistry, Faculty of Science, King Abdulaziz University, Jeddah, Saudi Arabia; 2 King Fahd Medical Research Center, King Abdulaziz University, Jeddah, Saudi Arabia; 3 Department of Chemistry, Faculty of Science, University of Bisha, Bisha, Saudi Arabia; 4 Department of Medical Laboratory Sciences, Faculty of Applied Medical Sciences, King Abdulaziz University, Jeddah, Saudi Arabia; 5 Special Toxicological Analysis Unit, Pathology and Laboratory Medicine DPLM, King Faisal Specialist Hospital and Research Center, Riyadh, Saudi Arabia; 6 Faculty of Medicine, Alfaisal University, Riyadh, Saudi Arabia; 7 Department of Natural Products, Faculty of Pharmacy, King Abdulaziz University, Jeddah, Saudi Arabia; 8 Department of Pharmaceutical Chemistry, Faculty of Pharmacy, King Abdulaziz University, Jeddah, Saudi Arabia

**Keywords:** catechin, GC-MS, kola nut, picropodophyllin, scopolin, yohimbine

## Abstract

**Introduction:**

Cancer continues to pose a critical global health challenge, with conventional therapies often limited by efficacy and adverse side effects. In search of safer and more effective treatments, medicinal plants offer a treasure trove of bioactive compounds with therapeutic potential. Kola nut (*Cola* spp.), a tropical evergreen native to the African rainforests, has long been valued in traditional medicine and as a natural stimulant.

**Methods:**

This study explores the anticancer potential of kola nut seeds extract by first identifying its chemical constituents through gas chromatography–mass spectrometry (GC-MS), followed by molecular docking of selected compounds against the cancer-associated target PI3Kα. A methanolic extract was fractionated using column chromatography, yielding nine distinct fractions. The first four fractions were analyzed directly by GC-MS, while the remaining five were derivatized (silylated) prior to analysis.

**Results and discussion:**

A total of 78 diverse compounds were identified, spanning alkaloids, flavonoids, fatty acids, and carbohydrates. From these, 15 bioactive compounds, including scopolin, picropodophyllin, catechin, yohimbine, squalene, campesterin, epicatechin, stigmasterol, *p*-coumaric acid, quinoline, methyl linoleate, methyl oleate, DTB-spdione, theobromine, and caffeine, were shortlisted for *in silico* docking studies. Molecular docking of 15 kola nut bioactive compounds against PI3Kα revealed that squalene, campesterin, epicatechin, yohimbine, and scopolin exhibited favorable binding energies, engaging key residues. Fatty-acid esters showed moderate binding, while small compounds such as theobromine and caffeine displayed weak interactions. Overall, these findings highlight the potential of kola nut phytochemicals as scaffolds for PI3Kα-targeted drug development. Based on enhanced fractionations, our study provides an expanded chemical profile of kola nut seeds and suggests that selected phytochemicals may serve as promising candidates for anticancer drug development. Further experimental validation is warranted to translate these insights into therapeutic applications.

## 1 Introduction

Cancer remains one of the most formidable global health challenges, with recent estimates reporting 19.3 million new cases and 10 million cancer-related deaths worldwide in 2020 (1). Alarmingly, cancer incidence rates are projected to increase substantially in the coming decades. Although advancements in therapeutic interventions have considerably improved patient survival, effective disease management remains challenging due to the complexity and aberrant regulation of signaling pathways. Among these pathways, the phosphoinositide 3-kinase (PI3K)/Akt/mammalian target of rapamycin (mTOR) pathway is particularly critical, as its dysregulation is frequently linked to tumor progression, resistance to therapy, and poor outcomes across multiple cancer types (2–4). The class I PI3K/Akt axis, one of the most commonly altered pathways in human cancers, comprises four heterodimeric isoforms (α, β, γ, and δ), each characterized by different expression patterns and specialized biological functions (5). Notably, the PI3Kα isoform demonstrates the highest frequency of activating mutations across different cancer types (6). Upon activation, PI3K catalyzes the phosphorylation of phosphatidylinositol-4,5-bisphosphate (PIP2) to generate phosphatidylinositol-3,4,5-trisphosphate (PIP3), a pivotal second messenger that initiates downstream signaling. As an upstream regulator of the Akt/mTOR cascade, PI3K plays a dynamic role in key cellular processes, including cell cycle progression, survival, proliferation, and actin cytoskeleton remodeling (7–10). This positions PI3K as an attractive target in the development of anticancer therapeutics. Phytochemicals, a class of naturally occurring plant-derived compounds with potent antioxidant properties, have demonstrated a wide range of anticancer activities (11–13). Kola nut, scientifically known as *Cola nitida*, is a tree belonging to the *Sterculiaceae* family, native to West Africa and cultivated in regions such as Brazil and India (14). The significance of the kola nut lies in its widespread use in the production of both carbonated and traditional beverages, as well as its role in folk medicine, reflecting its cultural heritage and value in the societies it originates from. The seeds have high caffeine content, which contributes to their use as a natural stimulant (15). In addition to caffeine, the seeds also contain a variety of other biologically active compounds, including alkaloids, tannins, and polyphenols (16). Recent studies have recognized several pharmacologically active constituents in kola nut seeds, such as theobromine, caffeine, and (+)-catechin, all of which demonstrate potential anticancer properties (17, 18). While conventional methods, including high-performance liquid chromatography (HPLC), have been employed to analyze specific compounds such as caffeine and crude protein (19, 20), a comprehensive phytochemical profile of kola nut seeds remains incomplete. The medicinal and economic significance of kola nut seeds has sparked growing interest in systematic phytochemical profiling and in exploring their anticancer potential. GC-MS has emerged as a powerful analytical tool for the detection and identification of bioactive compounds, such as steroids, terpenoids, alkaloids, flavonoids, tannins, phenols, and essential oils (21, 22). Simultaneously, advancements in computational structural biology have revolutionized drug discovery by enabling accurate prediction of ligand–target interactions and rational design of inhibitors (2, 3, 23–25). In this study, GC-MS is employed to elucidate the complete phytochemical composition of kola nut seeds, followed by the molecular docking of 15 selected bioactive compounds, including scopolin, picropodophyllin, catechin, yohimbine, squalene, campesterin, epicatechin, stigmasterol, *p*-coumaric acid, quinoline, methyl linoleate, methyl oleate, DTB-spdione, theobromine, and caffeine targeting PI3Kα, a critical oncogenic kinase implicated in cancer progression and therapeutic resistance. This integrated approach not only establishes the expanded chemical profile of kola nut seeds but also systematically evaluates their potential as a source of novel PI3Kα inhibitors for anticancer drug development. These findings provide a foundation for subsequent experimental validation and pharmacological exploration.

## 2 Materials and methods

### 2.1 Plant material and chemicals

Kola nut seeds were purchased from a local market in Jeddah, Saudi Arabia. The seeds were cleaned, freeze-dried, and finely ground using an electric grinder. The powdered sample was stored in an airtight container at 4 °C until further use. Hexane, dichloromethane (GC grade), and methanol (HPLC grade) were obtained from Fisher Scientific (Hampton, United States). Silica gel (60–120 mesh) for column chromatography was purchased from Laboratory Rasayan (India). N-Methyl-N-(trimethylsilyl) trifluoroacetamide (MSTFA, ≥98.5% purity) was sourced from Supelco^®^ (Germany).

### 2.2 Preparation of crude methanolic extract

The powdered seeds (72.31 g) were extracted using 500 mL of methanol for 72 h in a Soxhlet extractor, and the methanolic extract was then concentrated using a rotary evaporator and weighed to calculate the extraction yield percentage. The extraction yield percentage (%E. Y) was calculated using the following formula:


%E.Y=(Weight of extract yield/Initial weight of the sample)×100

### 2.3 Fractionization of crude methanolic extract

The crude methanolic extract was fractionated using column chromatography (80 cm × 3.5 cm), and silica gel served as the stationary phase, while *n*-hexane, dichloromethane, and methanol were sequentially applied as mobile phases in order of increasing polarity. The mobile phase gradient in the column chromatography began with *n*-hexane (non-polar), followed by dichloromethane (50, 70, and 100%). The initial fractions were collected and prepared for direct injection in GC-MS. Following that, the polarity was increased gradually using methanol (10, 20, and 30%), and the final fractions were collected and prepared for silylation reaction before GC-MS analysis. Thin-layer chromatography (TLC) was performed for all fractions to confirm the separation and presence of compounds. TLC spots were visualized under UV light at 254 and 366 nm and then by iodine crystal vapor. All fractions were concentrated after being collected using a rotary evaporator. A summary of the column chromatography conditions and corresponding fraction yields is provided in Table 1.

**Table 1:** Summary of fraction numbers, column chromatography conditions, and fraction yields.

Fraction number	Elution system (flow rate = 10 mL/min)	Yield %	Color
F1	Hexane 100%	0.5%	Light green
F2	Hexane: DCM (50:50)	0.85%	Green
F3	Hexane: DCM (50:50)	3.2%	Greenish yellow
F4	Hexane: DCM (30:70)	3.09%	Yellow
F5	DCM 100%	42.69%	White
F6	DCM: MeOH (90:10)	0.6%	Beige
F7	DCM: MeOH (90:10)	0.96%	Light brown
F8	DCM: MeOH (80:20)	5.34%	Light brown
F9	DCM: MeOH (70:30)	4.27%	Brown

### 2.4 Preparation of samples for gas chromatography analysis

#### 2.4.1 Direct injection

The first four fractions (100 mg each), were diluted with 100 μL hexane/dichloromethane, then transferred to 200 μL inserts of GC vials, and analyzed by GC-MS.

#### 2.4.2 Sialylation (derivatization) reaction

The non-polar fractions generally contain non-polar volatile compounds (such as alkanes and unsaturated fatty acids) that can be directly analyzed by GC-MS without derivatization. Conversely, the higher-polarity fractions require derivatization prior to GC-MS analysis to enhance their volatility and detectability. Therefore, the final dried fractions (100 mg each) were treated with 250 μL of N-methyl-N-(trimethylsilyl) trifluoroacetamide (MSTFA) before incubation at 80 °C for 20 min to ensure complete derivatization. The derivatized samples were cooled to room temperature, then diluted with 100 μL hexane, transferred to 200 μL inserts of GC vials, and analyzed by GC-MS.

### 2.5 Gas chromatography-mass spectrometry

The samples were analyzed using a Thermo Trace 1300 gas chromatograph coupled with a Thermo TSQ 8000 mass spectrometer (GC/MS). Separation was performed using a DB-5 fused silica capillary column (30 m length, 0.32 mm ID, and 0.25 μm film thickness) with helium as the carrier gas (flow rate: 1 mL/min; pressure: 13 psi). The oven was maintained at an initial temperature of 70 °C (held for 2 min), sequentially increased to 150 °C at 25 °C/min (held for 2 min), 200 °C at 3 °C/min (held for 6 min), and finally to 300 °C at 6.07 °C/min (held for 10 min). The ion source and MS transfer line temperatures were maintained at 250 °C and 290 °C, respectively, with an ionization voltage of 70 eV, and injection volume of 1 μL. Compound identification was executed by comparing retention times and mass spectra against reference standards available in the Wiley and NIST databases.

### 2.6 Data retrieval

The three-dimensional (3D) structure of PI3Kα in complex with its native inhibitor was collected from the Protein Data Bank (PDB ID: 4L23). Alpelisib (CID: 56649450) was selected as the positive control because it is an FDA-approved, highly selective PI3Kα inhibitor with well-established potency. The 3D structures of alpelisib and 15 bioactive compounds identified from kola nut seeds, such as squalene (CID: 638072), campesterin (CID: 173183), epicatechin (CID: 72276), yohimbine (CID: 8969), scopolin (CID: 439514), DTB-spdione (CID: 545303), picropodophyllin (CID: 72435), stigmasterol (CID: 5280794), *p*-coumaric acid (CID: 637542), catechin (CID: 9064), quinoline (CID: 7047), methyl linoleate (CID: 5284421), methyl oleate (CID: 5364509), theobromine (CID: 5429), and caffeine (CID: 2519), were retrieved from the PubChem database.

### 2.7 Molecular docking

Molecular docking of the four selected compounds into the active site of PI3Kα was performed using DOCK v6.9 (26). Initial preparation of the protein and ligands, including the addition of hydrogens and charge assignments, was carried out using UCSF Chimera v1.15 (27). The binding site was defined based on the position of the co-crystallized native inhibitor, and a region within 5 Å around the inhibitor was used as the docking grid.

### 2.8 Self-docking validation

To evaluate the accuracy of the docking protocol, a self-docking analysis was performed using the native co-crystallized inhibitor of PI3Kα. The native ligand bound in the crystal structure was extracted, prepared using the same protocol applied to all ligands, and subsequently re-docked into the PI3Kα binding site. The accuracy of pose reproduction was assessed by the root-mean-square deviation (RMSD) between the experimentally observed crystal pose and the re-docked pose in PyMOL v2.3.0 (28), employing the align command with both poses locked. RMSD values were computed for only heavy atoms, excluding hydrogen atoms, in accordance with standard docking validation practice.

### 2.9 Binding pose analysis

The binding poses of the docked compounds were analyzed and visualized using PyMOL v2.3.0 (28). Protein–ligand interaction diagrams were generated using LigPlot+ v2.2.8 (29), which also facilitated the measurement and annotation of hydrogen bond distances. Additionally, LigPlot+ provided an enumeration of the non-bonded contacts between the ligands and protein residues.

### 2.10 Binding energy and dissociation constant prediction

The binding energies and dissociation constants (pK_d_ values) were predicted using X-Score v1.2.11 (30). X-Score is an empirical scoring function that provides docking-independent estimates of ligand binding affinities toward the target protein.

## 3 Results and discussion

The methanolic crude extract of kola nut seeds yielded 12.96%. The crude extract was fractionated using column chromatography with a gradient of hexane and dichloromethane, leading to the separation of the first four fractions. Furthermore, the separation by column chromatography was continued using a gradient of dichloromethane and methanol, leading to the separation of the final five fractions. The chemical constituents of these nine fractions were analyzed by GC-MS.

The first four fractions, obtained using a hexane and dichloromethane gradient, were injected directly into the GC-MS. Approximately 39 compounds were identified from these fractions. The main compounds in the first fraction are 3,7-dimethyldecane (15.67%), tetracontane (13.88%), octadecane (8.78%), and 2,4-di-t-butyl-6-nitrophenol (7.19%). The major components in the second fraction are ethyl linoleate (23.29%), ethyl palmitate (14.24%), and ethyl oleate (11.38%).

In addition, the main compounds in the third fraction are 4-methoxy-2-methylbenzaldehyde (18.12%), DTB-spdione (14.63%), picropodophyllin (13.91%), and ethyl palmitate (12.82%). While the main compounds in the fourth fraction are stigmasterol (20.66%), caffeine (18.52%), hexanedioic acid, bis(2-ethylhexyl) (14.12%), and DTB-spdione (10.5%).

The last five fractions, obtained from a dichloromethane gradient with methanol, were derivatized (sialylated) before being injected into GC-MS. A total of 39 compounds were detected in these fractions. The major component in the fifth fraction is caffeine (81.59%), while the main compounds in the sixth fraction are octanedioic acid, 2TMS (34.19%) and azelaic acid, 2TMS (20.29%). In addition, the main compounds in the seventh and eighth fractions are catechin TMS (33.82, 41.29%, respectively) and epicatechin TMS (33.21, 39.65%, respectively). The main compounds in the ninth fraction are hexopyranose, 5TMS (33.0%) and d-glucopyranose, 5TMS (14.37%).

### 3.1 Selection of kola nut bioactive compounds for *in silico* study

**Figure 1:**
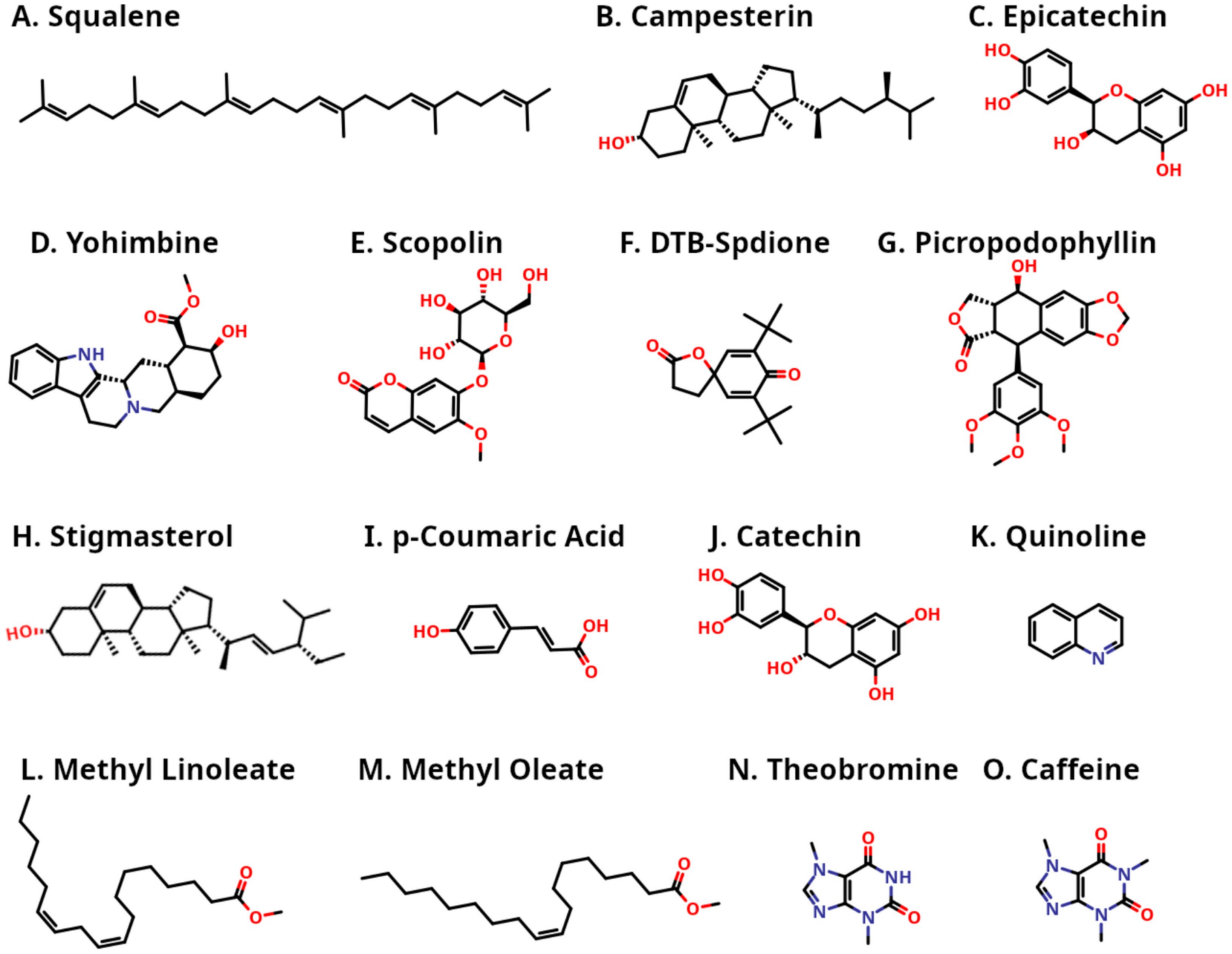
Two-dimensional chemical structures of the 15 selected kola nut bioactive compounds **(A-O)**. Heteroatoms are colored according to standard conventions: oxygen atoms (O) are shown in red and nitrogen atoms (N) are shown in blue, along with their attached hydrogens.

Finally, 15 bioactive compounds (see Figure 1) were selected from kola nut seeds based on their chemical features known to facilitate engagement with kinase binding pockets (31). These features include heterocyclic scaffolds capable of forming hinge-region hydrogen bonds (e.g., quinoline, caffeine, and theobromine), polar polyphenols that participate in hydrogen-bonding interactions (e.g., catechin, epicatechin, *p*-coumaric acid, and scopolin), and rigid polycyclic or alkaloid frameworks that provide shape complementarity within the ATP-binding site (e.g., picropodophyllin, yohimbine, and DTB-spdione). In addition, sterols and fatty-acid ester-like hydrophobic compounds (e.g., stigmasterol, campesterin, methyl oleate, methyl linoleate, and squalene) were included for their potential to occupy lipophilic sub pockets. All selected bioactive compounds have molecular weights below 500 Da and comply with most of Lipinski’s Rule of five criteria, supporting their potential drug-likeness. This selection strategy ensured representation of diverse chemical scaffolds capable of engaging PI3Kα through multiple interaction modes, including hydrophobic contacts, hydrogen bonding, and steric complementarity.

### 3.2 Self-docking validation

**Figure 2:**
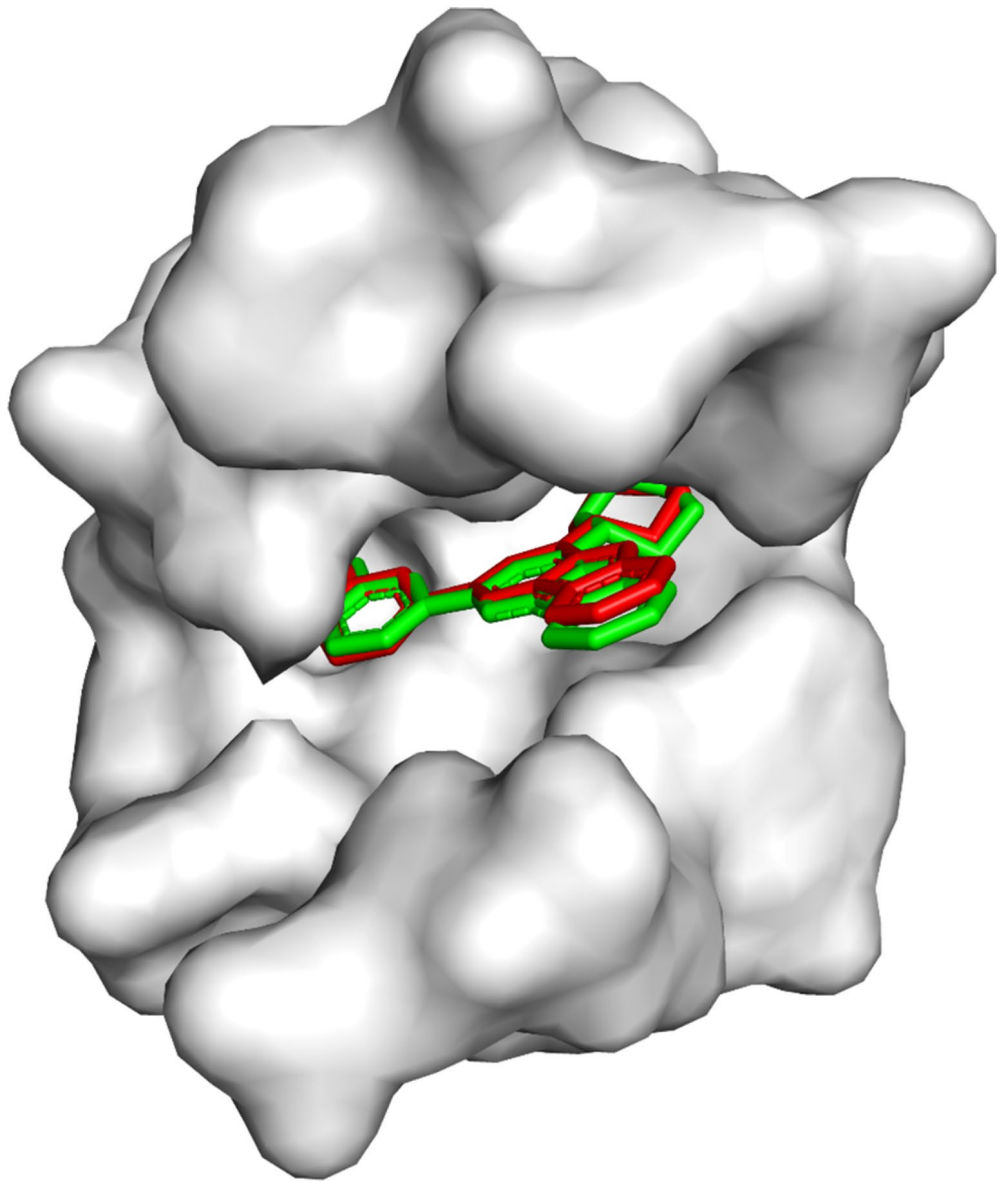
Superimposition of the native inhibitor in its crystallographic conformation (red) with the re-docked pose (green) in the active site. The near-perfect overlap indicates high fidelity of pose reproduction and validates the docking methodology.

The excellent overlap between the re-docked and crystallographic poses (Figure 2) visually confirms the accuracy of the docking protocol. To further quantify this agreement, RMSD evaluation was carried out using all 26 heavy atoms of the native inhibitor to quantify the agreement between the two poses. An RMSD value below 2.0 Å is widely accepted as the benchmark for a valid and reliable docking protocol. The RMSD obtained in this study was 0.187 Å, substantially lower than the accepted threshold, thereby indicating excellent pose reproducibility and high docking accuracy. This strong agreement further reinforces the reliability of the docking predictions generated for the screened cola nut bioactive compounds.

### 3.3 Molecular docking of kola nut bioactive compounds to the PI3Kα ATP-binding site

**Figure 3:**
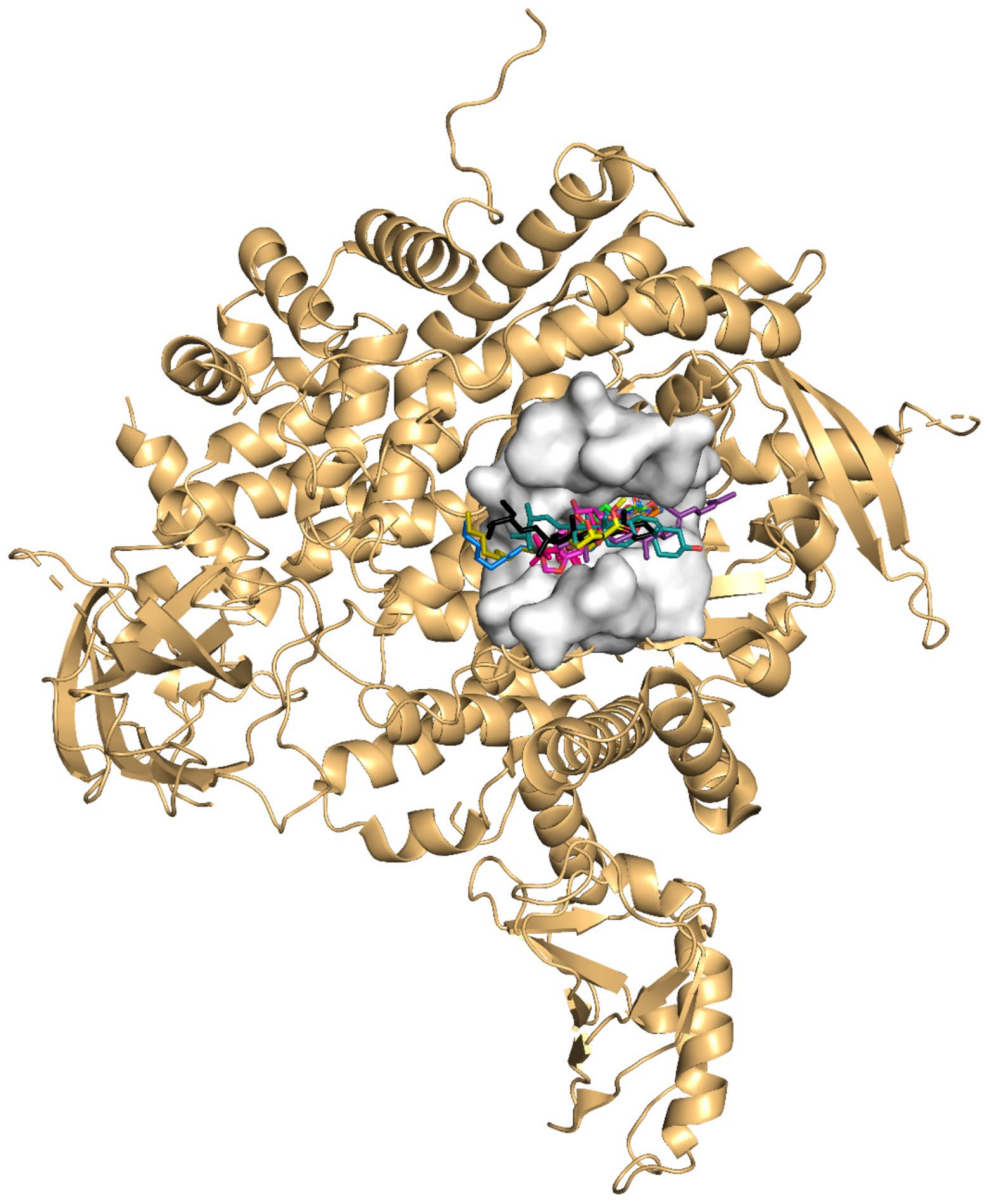
Ribbon representation of PI3Kα (light orange) with the ATP-binding site shown as a white surface. Docked compounds and the native inhibitor are shown in distinct colors to illustrate their binding poses. Overlapping orientations depict how the compounds are accommodated within the active site relative to the native ligand.

Molecular docking of 15 kola nut bioactive compounds against the ATP-binding pocket of PI3Kα revealed all of the compounds bound well within the pocket (Figure 3). Alpelisib served as an appropriate positive control as it is a clinically validated PI3Kα-selective inhibitor. Several compounds showed binding affinities comparable to the positive control alpelisib and the native inhibitor (Table 2). The native ligand exhibited a binding energy of −8.40 kcal/mol (pK_d_ 6.16), while alpelisib docked at −8.05 kcal/mol (pK_d_ 5.90). Among the screened bioactive compounds, squalene showed the most favorable binding energy (−8.22 kcal/mol, pK_d_ 6.03), surpassing that of alpelisib. The campesterin (−7.93 kcal/mol) and epicatechin (−7.83 kcal/mol) also showed binding affinities close to the positive control. Yohimbine, scopolin, DTB-spdione, picropodophyllin, stigmasterol, *p*-coumaric acid, and catechin displayed meaningful PI3Kα-binding potential, while quinoline, methyl linoleate, methyl oleate, theobromine, and caffeine exhibited comparatively weaker binding. Notably, methyl linoleate and methyl oleate exhibited modest binding energies but highly favorable dock scores, likely reflecting extensive surface contacts within the ATP-binding pocket. Caffeine and theobromine demonstrated the weakest binding, consistent with their small molecular size and limited hydrophobic surface area.

**Table 2:** Predicted binding energies, dissociation constants (pK_d_), and docking scores of selected kola nut bioactive compounds at the PI3Kα ATP-binding site.

Rank	CID	Name	Binding energy (kcal/mol)	pK_d_	Dock score
Reference	—	Native ligand	−8.40	6.16	−47.90
Reference	56,649,450	Alpelisib	−8.05	5.90	−40.62
1	638,072	Squalene	−8.22	6.03	−41.62
2	173,183	Campesterin	−7.93	5.81	−27.05
3	72,276	Epicatechin	−7.83	5.74	−34.91
4	8,969	Yohimbine	−7.71	5.65	−32.47
5	439,514	Scopolin	−7.59	5.57	−42.88
6	545,303	DTB-spdione	−7.56	5.54	−27.71
7	72,435	Picropodophyllin	−7.56	5.54	−37.58
8	5,280,794	Stigmasterol	−7.50	5.50	−27.55
9	637,542	*p*-Coumaric acid	−7.39	5.42	−27.58
10	9,064	Catechin	−7.31	5.36	−33.05
11	7,047	Quinoline	−7.23	5.30	−22.12
12	5,284,421	Methyl linoleate	−7.15	5.24	−44.06
13	5,364,509	Methyl oleate	−7.04	5.16	−45.00
14	5,429	Theobromine	−6.40	4.69	−28.80
15	2,519	Caffeine	−6.33	4.64	−28.18

The native bound ligand and the clinically approved PI3Kα inhibitor alpelisib are included for reference.

**Figure 4:**
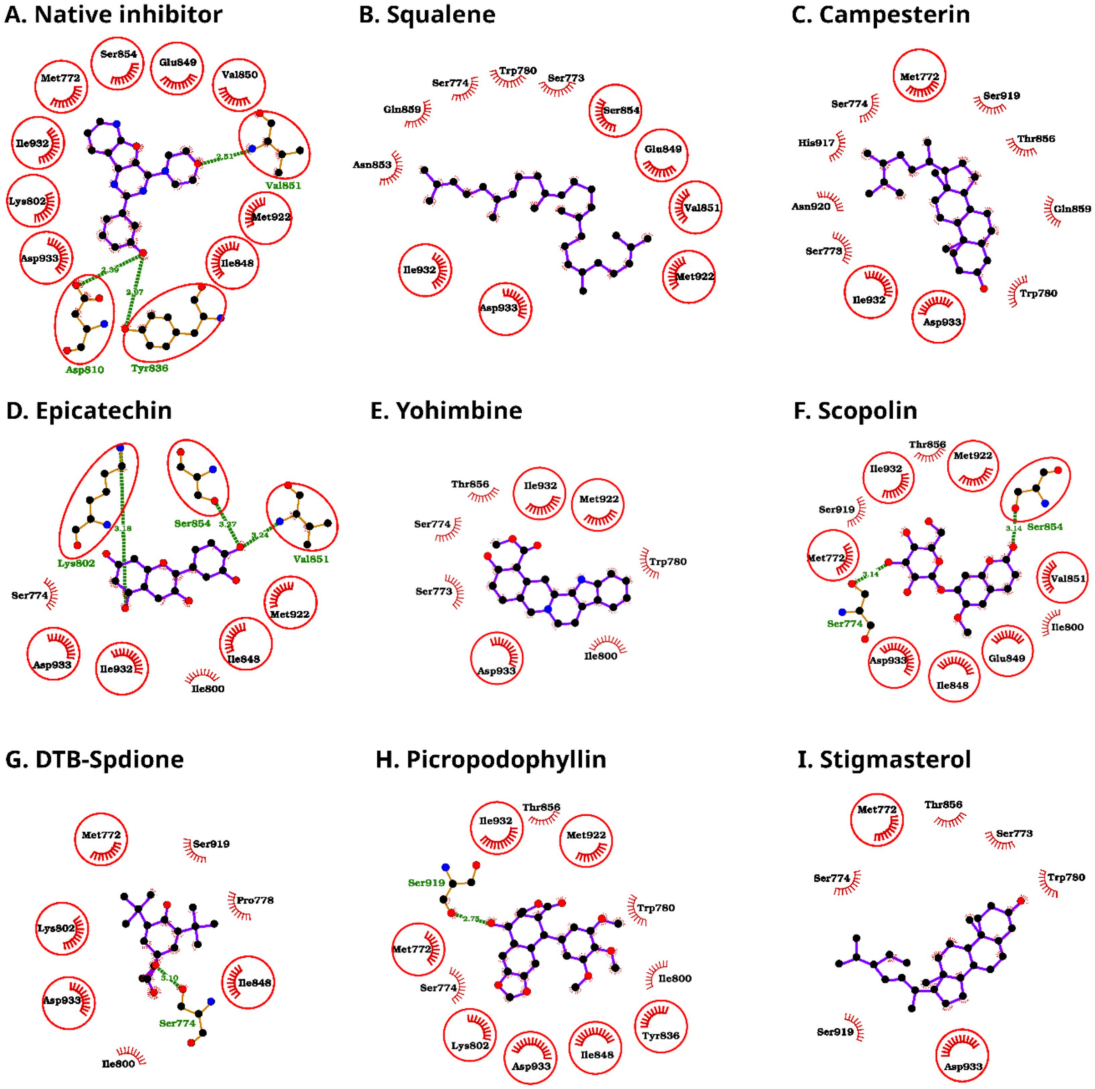
Protein-ligand interaction diagrams of the bound native inhibitor and the docked complexes of kola nut bioactive compounds with PI3Kα **(A-I)**. Hydrogen bonds are represented by green dashed lines with the corresponding bond lengths labeled (in Å), and hydrophobic (non-bonded) contacts are shown as spoked arcs around the ligand atoms, and the common residues having the native inhibitor binding capacity are additionally encircled for emphasis.

**Figure 5:**
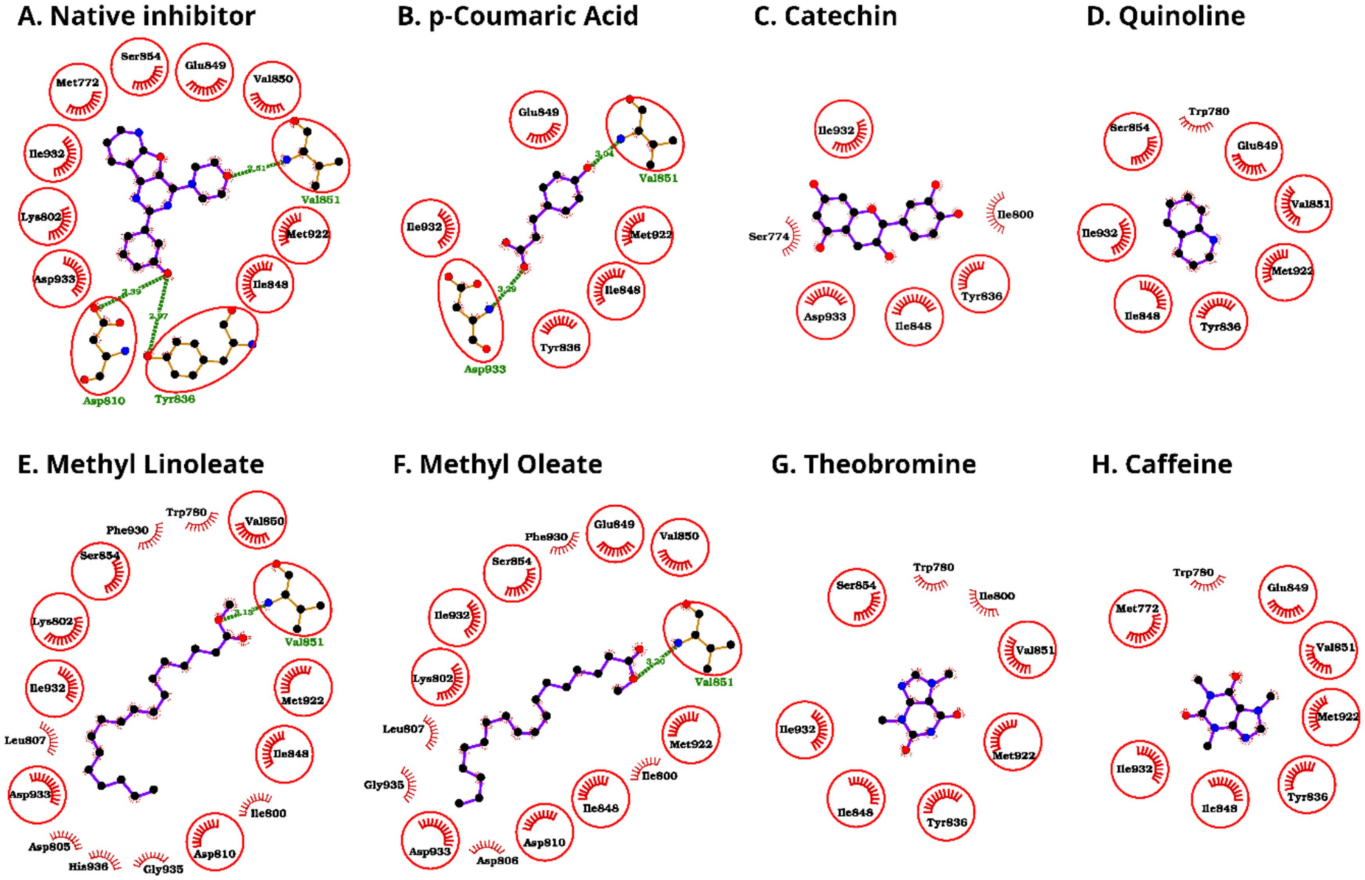
Protein-ligand interaction diagrams of the bound native inhibitor and the docked complexes of kola nut bioactive compounds with PI3Kα **(A-H)**. Hydrogen bonds are denoted by green dashed lines with the corresponding bond lengths labeled (in Å), and hydrophobic (non-bonded) contacts are shown as spoked arcs around the ligand atoms. The residues that are common with the native inhibitor binding pose are additionally encircled for emphasis.

Analysis of residue contacts revealed consistent engagement of several pocket-lining amino acids across diverse chemical scaffolds (Figures 4, 5). The amino acids Ile-932 and Asp-933 emerged as the most frequently contacted residues, with nearly all compounds forming multiple non-bonded interactions at these positions (Tables 3, 4). These residues showed exceptionally high contact counts; for example, Asp-933 reached 8 contacts with campesterin, 6 with *p*-coumaric acid and catechin, 11 with methyl linoleate, and 13 with methyl oleate, indicating their central role in stabilizing ligands regardless of scaffold type. Similarly, Ile-932 displayed high contact density, including seven contacts with catechin and theobromine, six with picropodophyllin, and five with *p*-coumaric acid and caffeine. Beyond these two hotspots, several other often contacted residues included Ser-774, Trp-780, Ile-800, Ile-848, Val-851, and Met-922. Although the number and distribution of contacts varied among ligands, most compounds interacted with a conserved cluster of residues in the ATP-binding site, indicating similar binding orientations despite chemical diversity. The compounds with stronger binding generally displayed extensive non-bonded contacts with Ile-932 and Asp-933, highlighting the importance of these two hotspot residues.

**Table 3:** Non-bonded contacts of kola nut bioactive compounds with PI3Kα residues.

Amino acid	Squalene	Campesterin	Epicatechin	Yohimbine	Scopolin	DTB-Spdione	Picropodophyllin	Stigmasterol
Met-772	—	1	—	—	1	1	2	2
Ser-773	2	2	—	3	—	—	—	2
Ser-774	3	1	1	3	1	6	6	3
Pro-778	—	—	—	—	—	4	—	—
Trp-780	4	1	—	3	—	—	2	2
Ile-800	—	—	2	3	2	1	4	—
Lys-802	—	—	1	—	—	6	3	—
Asp-805	—	—	—	—	—	—	—	—
Asp-806	—	—	—	—	—	—	—	—
Leu-807	—	—	—	—	—	—	—	—
Asp-810	—	—	—	—	—	—	—	—
Tyr-836	—	—	—	—	—	—	2	—
Ile-848	—	—	1	—	3	1	4	—
Glu-849	1	—	—	—	2	—	—	—
Val-850	—	—	—	—	—	—	—	—
Val-851	1	—	2	—	1	—	—	—
Asn-853	3	—	—	—	—	—	—	—
Ser-854	5	—	—	—	—	—	—	—
Thr-856	—	1	—	1	1	—	1	1
Gln-859	5	3	—	—	—	—	—	—
His-917	—	1	—	—	—	—	—	—
Ser-919	—	2	—	—	3	1	3	1
Asn-920	—	1	—	—	—	—	—	—
Met-922	1	—	3	2	3	—	2	—
Phe-930	—	—	—	—	—	—	—	—
Ile-932	3	1	3	4	4	—	6	—
Asp-933	3	8	2	2	4	5	5	3
Gly-935	—	—	—	—	—	—	—	—
His-936	—	—	—	—	—	—	—	—

Numbers indicate the count of non-bonded interactions between each compound and the listed amino acids.

**Table 4:** Non-bonded contacts of kola nut bioactive compounds with PI3Kα residues.

Amino acid	*p*-Coumaric acid	Catechin	Quinoline	Methyl linoleate	Methyl oleate	Theobromine	Caffeine
Met-772	—	—	—	—	—	—	2
Ser-773	—	—	—	—	—	—	—
Ser-774	—	2	—	—	—	—	—
Pro-778	—	—	—	—	—	—	—
Trp-780	—	—	3	1	—	2	1
Ile-800	—	4	—	2	1	2	—
Lys-802	—	—	—	5	4	—	—
Asp-805	—	—	—	2	—	—	—
Asp-806	—	—	—	—	1	—	—
Leu-807	—	—	—	1	2	—	—
Asp-810	—	—	—	3	3	—	—
Tyr-836	1	1	6	—	—	5	4
Ile-848	4	2	1	4	1	3	2
Glu-849	1	—	1	—	1	—	1
Val-850	—	—	—	3	3	—	—
Val-851	2	—	3	2	2	3	2
Asn-853	—	—	—	—	—	—	—
Ser-854	—	—	2	1	1	1	—
Thr-856	—	—	—	—	—	—	—
Gln-859	—	—	—	—	—	—	—
His-917	—	—	—	—	—	—	—
Ser-919	—	—	—	—	—	—	—
Asn-920	—	—	—	—	—	—	—
Met-922	2	—	4	2	1	2	4
Phe-930	—	—	—	2	2	—	—
Ile-932	5	7	3	1	1	7	5
Asp-933	6	6	—	11	13	—	—
Gly-935	—	—	—	1	1	—	—
His-936	—	—	—	2	—	—	—

Numbers indicate the count of non-bonded interactions between each compound and the listed amino acids.

Hydrogen-bond analysis revealed that several compounds contributed to the stabilization of polar interactions (Table 5 and Figures 4, 5). Epicatechin formed three hydrogen bonds with Lys-802, Val-851, and Ser-854; scopolin formed two with Ser-774 and Ser-854; and *p*-coumaric acid formed two with Val-851 and Asp-933. Fatty-acid esters like methyl linoleate and methyl oleate formed single hydrogen bonds with Val-851. Picropodophyllin and DTB-spdione each formed one hydrogen bond with Ser-919 and Ser-774, respectively. Conversely, purely hydrophobic compounds like squalene, campesterin, and stigmasterol lacked hydrogen bonds but still achieved competitive binding energies via the presence of non-bonded interactions. Theobromine and caffeine showed weak binding, forming only a few non-bonded contacts and no hydrogen bonds. Their small molecular size, limited aromatic surface, and low lipophilicity prevent them from occupying and stabilizing the ATP-binding pocket effectively.

**Table 5:** Hydrogen-bond interactions of kola nut bioactive compounds with PI3Kα.

Compounds	No. of H-bonds	Interacting residues (bond length, Å)
Squalene	0	—
Campesterin	0	—
Epicatechin	3	Lys-802 (3.18), Val-851 (3.24), Ser-854 (3.27)
Yohimbine	0	—
Scopolin	2	Ser-774 (3.14), Ser-854 (3.14)
DTB-SPDIONE	1	Ser-774 (3.10)
Picropodophyllin	1	Ser-919 (2.75)
Stigmasterol	0	—
*p*-Coumaric acid	2	Val-851 (3.04), Asp-933 (3.29)
Catechin	0	—
Quinoline	0	—
Methyl linoleate	1	Val-851 (3.13)
Methyl oleate	1	Val-851 (3.20)
Theobromine	0	—
Caffeine	0	—

Number of hydrogen bonds for each compound and the residues involved with bond lengths (Å) are shown.

The combination of strong hydrophobic complementarity, selective hydrogen bonding, and interaction with key hotspot residues demonstrate that several kola nut bioactive compounds represent promising scaffolds for PI3Kα inhibitor development. The diversity of chemical scaffolds, including hydrophobic terpenoids and sterols, flavonoids, alkaloids, coumaric-acid derivatives, and fatty-acid esters, recommends multiple avenues for structure-based optimization and potential lead-compound design. Overall, these results emphasize the potential of kola nut bioactive compounds as starting points for PI3Kα-targeted drug discovery and offer structural insights that can guide future optimization strategies.

**Figure 6:**
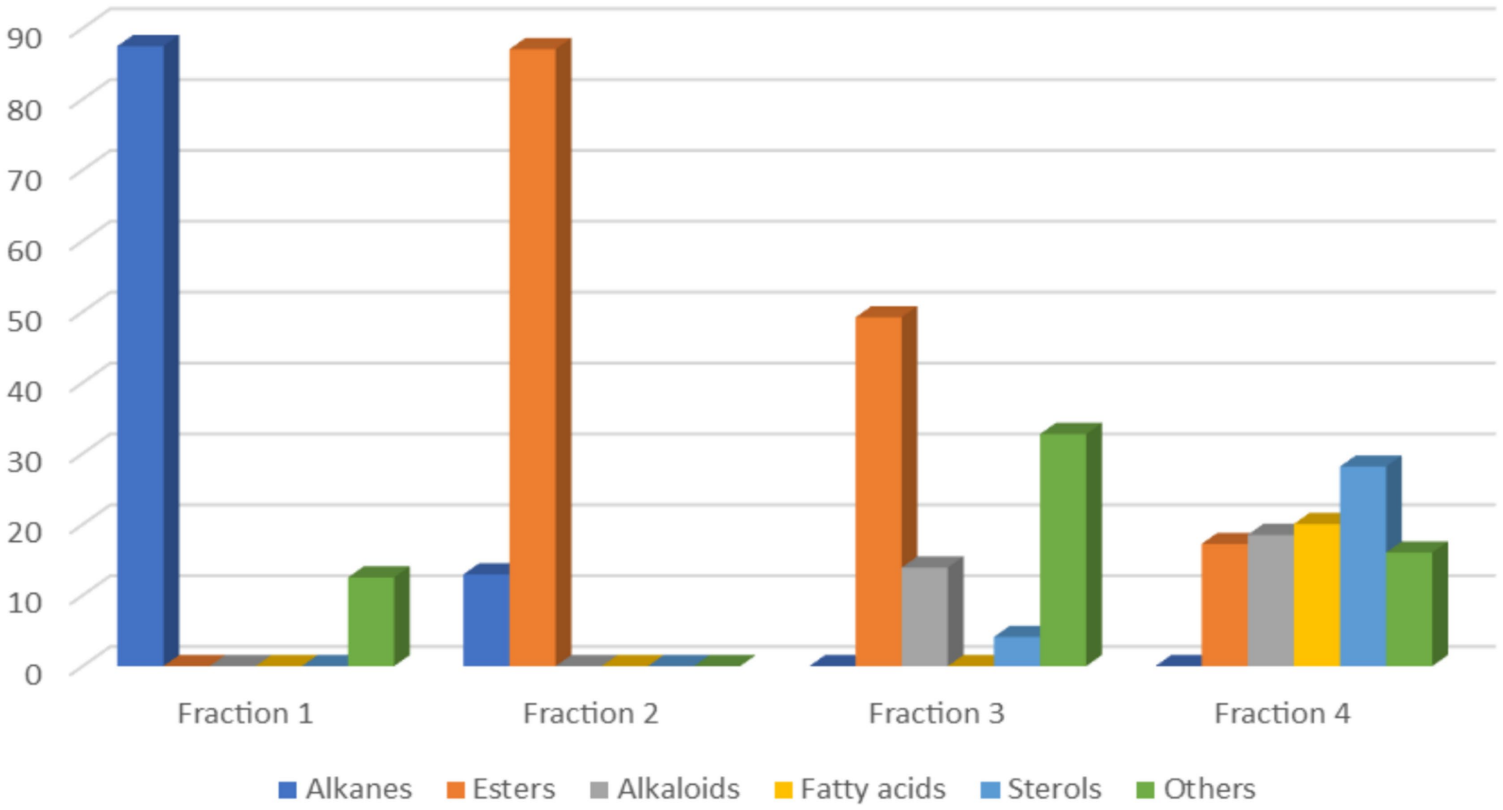
Histogram of chemical classes of compounds detected in the first four fractions.

GC-MS analysis revealed approximately 39 composites present in the first four fractions. The composition of these fractions was categorized as follows: alkanes constituted 22.92%, esters (including fatty acid esters) accounted for 24.2%, fatty acids constituted 8.27%, sterol accounted for 12.87%, and alkaloids accounted for 11.89%, while other compounds comprised approximately 19.86%. Figure 6 illustrates the relative proportions of each chemical class across the first four fractions.

In the first fraction, 16 compounds were identified, mainly consisting of alkanes with 87.48%. Notably, 3,7-dimethyldecane emerged as the most abundant compound in this fraction, representing 15.67% of the total, followed by tetracontane at 13.88%.

The second fraction contained 13 identified compounds, with 12.95% being linear saturated hydrocarbons and 87.05% comprising fatty acid esters. The principal compounds in this fraction included ethyl linoleate (23.29%), ethyl palmitate (14.24%), and ethyl oleate (11.38%).

In the third fraction, nine compounds were documented. 4-Methoxy-2-methylbenzaldehyde being the most abundant at 18.12%, followed by DTB-spdione at 14.63%, followed by picropodophyllin at 13.91%. Finally, the fourth fraction consisted of 11 identified compounds. Stigmasterol (20.66%), caffeine (18.52%), and hexanedioic acid, bis(2-ethylhexyl) (14.12%) being the most abundant.

**Figure 7:**
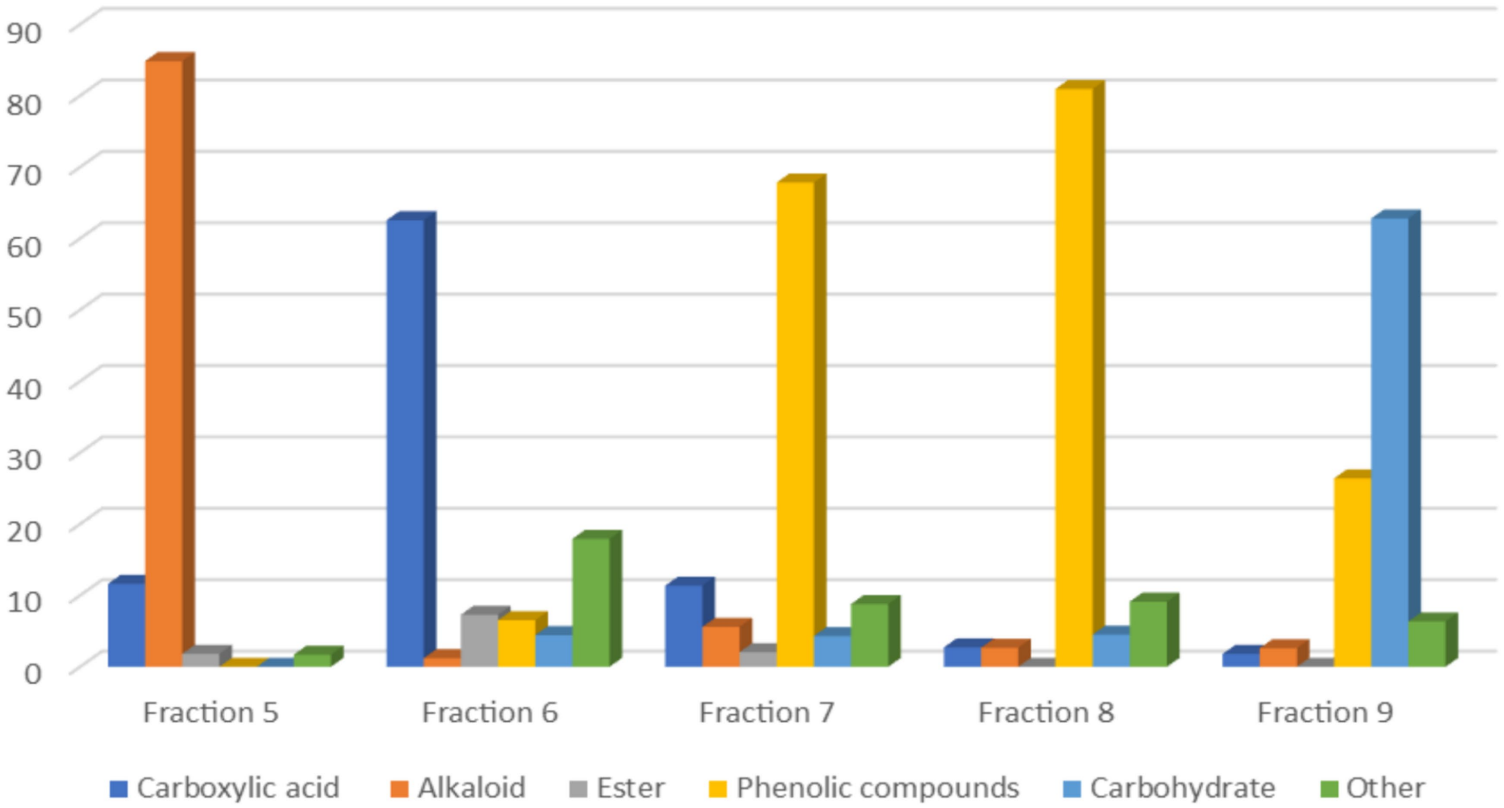
Histogram of chemical classes of compounds detected in the last five fractions.

Silylation procedure is frequently used to derivatize non-volatile compounds, like carboxylic acids, phenols, or alcohols by replacing a hydrogen atom in the hydroxyl gruops with a trimethylsilyl (TMS) group (32). The last separated fractions (fractions 5–9) were derivatized to conduct the analyses for non-polar and polar components using GC-MS. The chemical compounds were identified and classified into six main groups: carboxylic acids (18.02%), alkaloids (19.39%), esters (2.24%), phenolic compounds (36.34%), carbohydrates (15.22%), and other compounds (8.78%). Figure 7 illustrates the relative proportions of each chemical class across the last five fractions.

The fifth fraction was primarily composed of alkaloids (84.85%), primarily caffeine, with minor contributions from carboxylic acids (11.63%) and trace amounts of esters (1.82%). In contrast, the sixth fraction showed a high concentration of carboxylic acids, particularly fatty acids (62.56%), along with fatty acid esters (7.31%), phenolic compounds (6.53%), and phosphoric acid (17.93%).

The seventh and eighth fractions were characterized with high phenolic content of 67.85 and 80.94%, respectively, accompanied by varying levels of carboxylic acids, alkaloids, and carbohydrates. Notably, the eighth fraction contained the highest percentage of phenolic compounds among all fractions.

The ninth fraction displayed a distinct carbohydrate-rich profile (62.81%), complemented by a substantial phenolic component (26.39%) and minor alkaloid and carboxylic acid constituents. This distribution pattern across fractions depicts the efficacy of the chromatographic separation in isolating compounds based on their chemical properties and polarities.

The separation of methanolic kola nut seed extract by column chromatography demonstrated high competence, yielding nine fractions that contained the extract’s individual components. The distribution of compounds across these fractions was dependent on the polarity. The non-polar compounds concentrated in the first fractions (1–4), medium-polar compounds in the middle fractions (5–7), and highly polar compounds in the last fractions (8, 9). This distribution reflects the clear relationship between the polarity of the compounds and their exit order from the chromatographic column, where more polar compounds interact more strongly with the stationary phase (silica gel), leading to a delay in elution. The use of a solvent gradient from hexane (non-polar) to dichloromethane and then methanol (polar) contributed to improving the separation efficiency, allowing the separation of compounds based on their polarity differences.

Previous studies have shown analyses of kola nut seed extracts using GC-MS, and their findings align with those of the present study. Specifically, Suhail et al. (13) identified caffeine, stigmasterol, catechin, squalene, and some fatty acids and sugars in their analysis, which matches the results of this study. Furthermore, Salahdeen et al. (33) reported the presence of octadecane, caffeine, methyl palmitate, methyl linoleate, stigmasterol, and linoleic acid ethyl ester, all of which were also detected in this study. These findings underscore the dependability of the compounds identified across different studies. The results showed the presence of several compounds previously reported to exhibit significant biological activities. Specifically, octacosane (34), 1-octadecanol (35), docosane (36), heneicosane (37), *p*-coumaric acid (38), nonanoic acid, octadecane-9-enoic acid, and 18-nonadecenoic acid (39) have been reported in previous studies to exhibit antimicrobial activity. Furthermore, caffeine (40), ethyl linoleate (41), and nonacosane (36) have been identified in prior research for their anti-inflammatory properties. Additionally, compounds such as picropodophyllin (42), stigmasterol (43), campesterin (41), eicosane (44), tetracosane (36), methyl palmitate (45), methyl linoleate (46), yohimbine (47), squalene (48), scopoline (49) and catechin (50) have proved potential efficacy in treating cancers. These findings underscore the phamacological relevance of these compounds and highlight their potential therapeutic applications. Table 6 illustrates the biological activities of some compounds isolated from the fractions separated from the methanolic extract.

**Table 6:** Biological activity of some compounds identified in the fractionated methanolic extract.

No.	Compound name	Compound nature	Biological activity
1	Nonanoic acid	Fatty acid	Antimicrobial activity (40)
2	Tetradecane	Alkane	Antifungal and antimicrobial activities (44, 55)
3	Hexadecane	Alkane	Antifungal, antibacterial, and antioxidant activities (44)
4	2,4-Di-t-butyl-6-nitrophenol	Phenol	Antioxidant activity (46)
5	Octadecane	Alkane	Antifungal, antioxidant, and anti-inflammatory activities (36, 56)
6	1-Octadecanol	Alcohol	Antimicrobial activity (35)
7	2-Pentadecanone, 6,10,14-trimethyl-	Ketone	Antibacterial activity (57)
8	Caffeine	Alkaloid	Anti-inflammatory activity (40)
9	Methyl palmitate	Fatty acid ester	Antioxidant, anticancer, antifungal, antibacterial and anti-inflammatory activities (41, 44, 45, 58)
10	DTB-spdione	Ketone	Antimicrobial, and antifungal activity (55)
11	Ethyl palmitate	Fatty acid ester	Antioxidant, antimicrobial, and anti-inflammatory activities (57, 58)
12	Eicosane	Alkane	Antitumor, antimicrobial, antifungal, antidiabetic and antioxidant activities (36, 44, 55, 56)
13	Methyl linoleate	Fatty acid ester	Antioxidant, antimicrobial, anticancer, and anti-inflammatory activities (41, 46, 57)
14	Methyl oleate	Fatty acid ester	Antioxidant, antimicrobial, and anticancer activities (59)
15	Ethyl linoleate	Fatty acid ester	Anti-inflammatory activity (41)
16	Octadec-9-enoic acid	Fatty acid	Antimicrobial activity (39)
17	Docosane	Alkane	Antibacterial activity (36)
18	18-Nonadecenoic acid	Fatty acid	Antimicrobial activity (39)
19	Heneicosane	Alkane	Antimicrobial activity (37)
20	Tetracosane	Alkane	Anti-cancer, antioxidant, and antibacterial activities (34, 36, 56)
21	Hexanedioic acid, bis(2-ethylhexyl) ester	Ester	Antimicrobial activity (60)
22	Octacosane	Alkane	Antimicrobial activity (34)
23	Nonacosane	Alkane	Antibacterial and anti-inflammatory activities (36)
24	Squalene	Alkane	Antioxidant, antitumor, and cholesterol-lowering activities (48, 55)
25	Campesterin	Sterol	For the prevention of cardio metabolic diseases, and anticancer activities (43)
26	Tetracontane	Alkane	Anti-inflammatory activity (55)
27	Stigmasterol	Sterol	Anticancer; anti-inflammatory, antioxidant, anti-diabetic and cholesterol-lowering activities (43, 57)
28	Picropodophyllin	Alkaloid	Anticancer activity (42)
29	Quinoline	Alkaloid	Antimicrobial, anticancer, and anti-inflammatory activities (61)
30	Lactic acid	Carboxylic acid	Antimicrobial (38)
31	Catechin	Flavonoids	Antioxidant (13, 50)
32	Epicatechin	Flavonoids	Antioxidant (50)
33	Theobromine	Alkaloid	Antitumoral and anti-inflammatory activities (62)
34	Octadecanoic acid	Fatty acid	Antimicrobial (38)
35	Octanedioic acid	Fatty acid	Antimicrobial (38)
36	Yohimbine	Indole alkaloid	Anti-inflammatory and anticancer activities (47)
37	Glycerol	Polyol	Antimicrobial (38)
38	*p*-Coumaric acid	Phenolic acid	Antioxidant and antimicrobial activities (63)
39	Scopelin	Coumarin derivative	Antioxidant (64)

Although kola nut contains several key phytochemicals relevant to novel drug discovery (16, 51), both *Cola nitida* and *Cola acuminata* share many of the same bioactive compounds, with only minor differences in specific compounds. Therefore, these species-specific variations in phytochemical composition should be considered when assessing potential off-target risks. For example, both *C. nitida* and *C. acuminata* contain substantial amounts of caffeine and theobromine, which exert stimulant effects. Consequently, high or prolonged consumption of these nuts may lead to several adverse effects, including vomiting, tachycardia, anxiety, headaches, irregular heartbeat, and difficulty sleeping, primarily due to their high caffeine content (52, 53). Thus, negative effects associated with excessive caffeine intake require special attention, especially in children and pregnant women. A previous study suggested that patients with ulcers should avoid consuming *C. acuminata* due to its high acidic amino acid levels (54). Therefore, further research should conduct a comparative analysis of the bioactive compounds in both species and also assess their respective biological activities.

## 4 Conclusion

The analysis of nine fractions isolated from the methanolic extract of kola nut seeds using GC-MS revealed a diverse array of bioactive compounds, including caffeine, theobromine, stigmasterol, catechin, epicatechin, squalene, yohimbine, DTB-spdione, and several fatty acid esters. The presence of these compounds highlights the potential antimicrobial, anti-inflammatory, and anticancer properties of kola nut seeds. Among the 78 identified bioactive compounds, 15 were selected for evaluation of potential PI3Kα kinase inhibition. Computational analysis demonstrated that squalene exhibited the most favorable binding energy (−8.22 kcal/mol), followed by campesterin (−7.93 kcal/mol), epicatechin (−7.83 kcal/mol), yohimbine (−7.71 kcal/mol), scopolin (−7.59 kcal/mol), picropodophyllin (−7.56 kcal/mol), and other compounds. These compounds form extensive contacts with key hotspot residues Ile-932 and Asp-933. Fatty-acid esters such as methyl linoleate and methyl oleate exhibited moderate binding energies with favorable docking scores, while smaller molecules such as theobromine and caffeine showed weaker interactions due to limited size and lipophilicity. Collectively, these preliminary findings suggest that all 15 compounds may act as potential PI3Kα inhibitors for the management and treatment of various cancers. Furthermore, experimental validation is essential to translate these insights into viable therapeutic applications.

## Data Availability

The datasets presented in this study can be found in online repositories. The names of the repository/repositories and accession number(s) can be found in the article/Supplementary material.
