# Supplementary material for: Unveiling bioactive compounds in kola nut seeds: GC-MS identification and computational analysis for anticancer potential
Source: Front Nutr. 2026 Jan 9;12:1706709. doi: 10.3389/fnut.2025.1706709 (PMC12827131; doi:10.3389/fnut.2025.1706709)
Supplement: Supplementary file 1 [file Table_1.docx]

**Appendix**

The chromatograms of the first fractions (1, 2, 3, and 4) are shown in Figure (1). Table (1) provides detailed information about the components of the first four fractions.

| 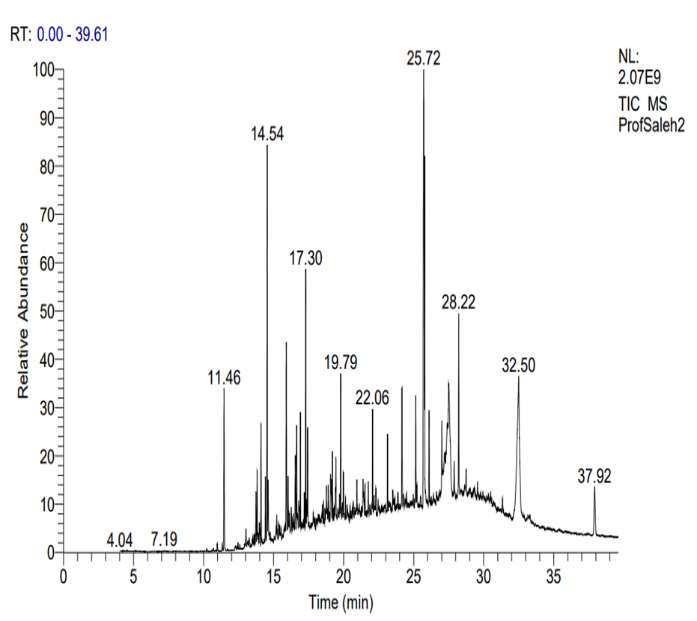 | | 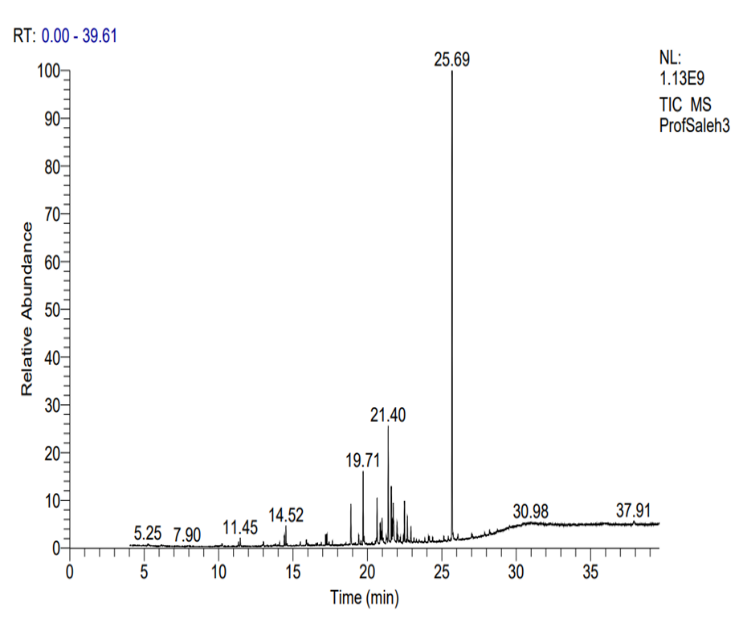 | | |
| --- | --- | --- | --- | --- |
| (**a**) | | (**b**) |  |  |
| 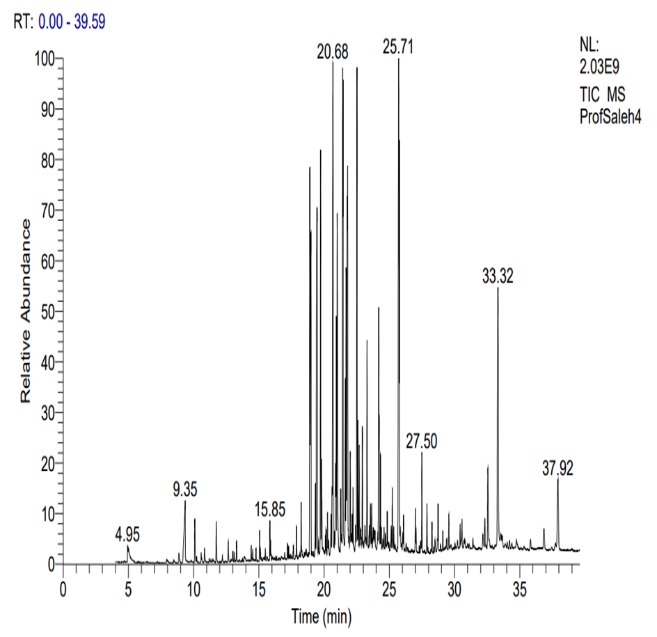 | 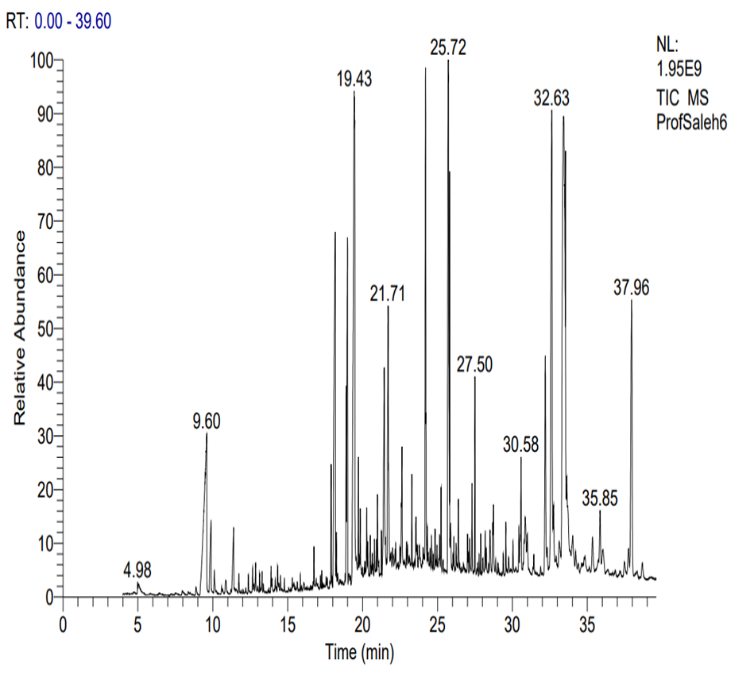 | | |  |
| (**c**) | (**d**) | | |  |

**Figure (1).** GC-MS chromatograms of fraction 1 (a), and fraction 2 (b), fraction 3 (c), and fraction 4 (d) of methanolic extract.

**Table (1): GC-MS results of the first four** **fractions separated from methanolic extract**

|  | **t_R_** | **Compound name** | **Molecular formula** | **Molecular mass(g/mol)** | **Area%** | **SI** |
| --- | --- | --- | --- | --- | --- | --- |
| **Fraction 1** | 11.46 | Tetradecane | C_14_H_30_ | 198 | 7.05 | 764 |
|  | 14.10 | 3-Methylpentadecane | C_16_H_34_ | 226 | 3.94 | 917 |
|  | 14.54 | 3,7-Dimethyldecane | C_12_H_26_ | 170 | 15.67 | 801 |
|  | 15.90 | 2,4-Di-t-butyl-6-nitrophenol | C_14_H_21_NO_3_ | 251 | 7.19 | 905 |
|  | 16.55 | 7-Methylheptadecane | C_18_H_38_ | 254 | 3.33 | 726 |
|  | 16.65 | 4-Methylhexadecane | C_17_H_36_ | 240 | 3.63 | 846 |
|  | 16.91 | 3-Methylheptadecane | C_18_H_38_ | 254 | 3.47 | 927 |
|  | 17.29 | Octadecane | C_18_H_38_ | 254 | 8.78 | 830 |
|  | 17.43 | 1-Octadecanol | C_18_H_38_O | 270 | 5.33 | 722 |
|  | 19.79 | Eicosane | C_20_H_42_ | 282 | 4.74 | 949 |
|  | 22.06 | Docosane | C_22_H_46_ | 310 | 3.65 | 922 |
|  | 24.17 | Tetracosane | C_24_H_50_ | 338 | 5.06 | 853 |
|  | 25.15 | Octacosane | C_28_H_58_ | 394 | 4.43 | 930 |
|  | 26.10 | Nonacosane | C_29_H_60_ | 408 | 3.27 | 899 |
|  | 28.22 | Squalene | C_30_H_50_ | 410 | 6.57 | 915 |
|  | 32.50 | Tetracontane | C_40_H_82_ | 562 | 13.88 | 736 |
| **Fraction 2** | 11.45 | Tetradecane | C_14_H_30_ | 198 | 3.01 | 884 |
|  | 14.52 | Hexadecane | C_16_H_34_ | 226 | 4.16 | 935 |
|  | 17.28 | Octadecane | C_18_H_38_ | 254 | 3.26 | 921 |
|  | 18.89 | Methyl palmitate | C_17_H_34_O_2_ | 270 | 8.69 | 899 |
|  | 19.71 | Ethyl palmitate | C_18_H_36_O_2_ | 284 | 14.24 | 924 |
|  | 20.65 | 8,11-Octadecadienoic acid, methyl ester | C_19_H_34_O_2_ | 294 | 11.14 | 811 |
|  | 20.98 | Methyl oleate | C_19_H_36_O_2_ | 296 | 6.65 | 898 |
|  | 21.26 | Methyl octadecenoate | C_19_H_38_O_2_ | 298 | 3.18 | 759 |
|  | 21.40 | Ethyl linoleate | C_20_H_36_O_2_ | 308 | 23.29 | 807 |
|  | 21.67 | 9,12-Octadecadienoic acid, ethyl ester | C_20_H_36_O_2_ | 308 | 2.85 | 872 |
|  | 21.73 | Ethyl oleate | C_20_H_38_O_2_ | 310 | 11.38 | 838 |
|  | 22.69 | 8,11-Eicosadienoic acid, methyl ester | C_21_H_38_O_2_ | 322 | 5.62 | 774 |
|  | 24.15 | Heneicosane | C_21_H_44_ | 296 | 2.52 | 746 |
| **Fraction 3** | 18.91 | Methyl palmitate | C_17_H_34_O_2_ | 270 | 11.08 | 916 |
|  | 18.99 | 7,9-Di-tert-butyl-1-oxaspiro(4,5)deca-6 ,9-diene-2,8-dione | C_17_H_24_O_3_ | 276 | 14.63 | 714 |
|  | 19.45 | 4-Methoxy-2-methylbenzaldehyde | C_9_H_10_O_2_ | 150 | 18.12 | 892 |
|  | 19.73 | Ethyl palmitate | C_18_H_36_O_2_ | 284 | 12.82 | 701 |
|  | 20.93 | Linoleic acid methyl ester | C_19_H_34_O_2_ | 294 | 8.62 | 921 |
|  | 21.69 | Linoleic acid ethyl ester | C_20_H_36_O_2_ | 308 | 6.05 | 895 |
|  | 24.19 | Hexanedioic acid, bis(2-ethylhexyl) ester | C_22_H_42_O_4_ | 370 | 10.65 | 887 |
|  | 32.55 | Stigmasterol | C_29_H_48_O | 412 | 4.12 | 701 |
|  | 33.32 | Picropodophyllin | C_22_H_22_O_8_ | 414 | 13.91 | 837 |
| **Fraction 4** | 9.58 | Nonanoic acid | C_9_H_18_O_2_ | 158 | 8.92 | 708 |
|  | 17.90 | 2-Pentadecanone, 6,10,14-trimethyl- | C_18_H_36_O | 268 | 2.54 | 931 |
|  | 18.16 | Caffeine | C_8_H_10_N_2_O_2_ | 194 | 18.52 | 823 |
|  | 18.90 | Methyl palmitate | C_17_H_34_O_2_ | 270 | 3.11 | 916 |
|  | 18.99 | 7,9-Di-tert-butyl-1-oxaspiro(4,5)deca-6,9-diene-2,8-dione | C_17_H_24_O_3_ | 276 | 10.5 | 639 |
|  | 21.44 | Octadec-9-enoic acid | C_18_H_34_O_2_ | 282 | 7.7 | 805 |
|  | 22.63 | 18-Nonadecenoic acid | C_19_H_36_O_2_ | 296 | 3.44 | 831 |
|  | 24.20 | Hexanedioic acid, bis(2-ethylhexyl) ester | C_22_H_42_O_4_ | 370 | 14.12 | 751 |
|  | 30.86 | 2(1H)-pyrimidinone, 3,4-dihydro-3,5-diphenyl-4-(phenylimino)-6-(phenylmethyl) -1-(2,4,6-rimethylphenyl)- | C_38_H_33_N_3_O | 547 | 2.98 | 896 |
|  | 32.19 | Campesterin | C_28_H_48_O | 400 | 7.51 | 724 |
|  | 32.63 | Stigmasterol | C_29_H_48_O | 412 | 20.66 | 632 |

**SI: match factor**

**t_R_: Retention time**

The chromatograms of the last fractions (5, 6, 7, 8, and 9) are shown in Figure (2). Table (2) provides detailed information about the compounds of the last five fractions.

| 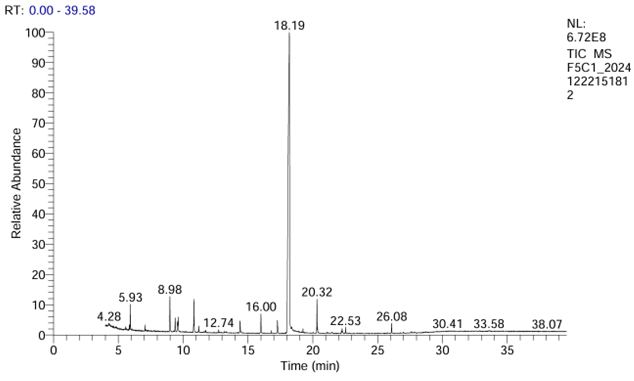 | | 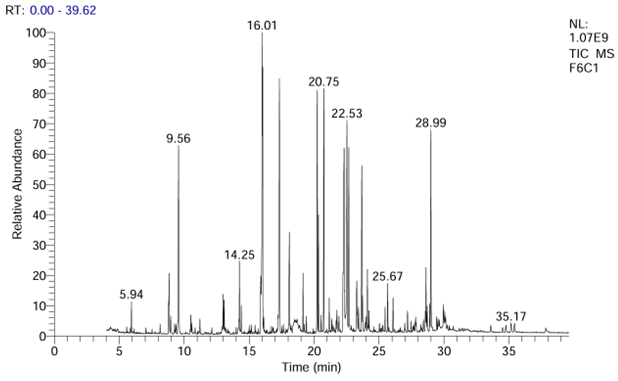 |
| --- | --- | --- |
| (**a**) | | (**b**) |
| 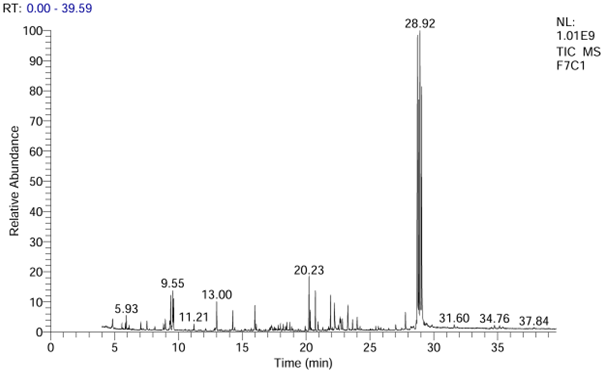 | 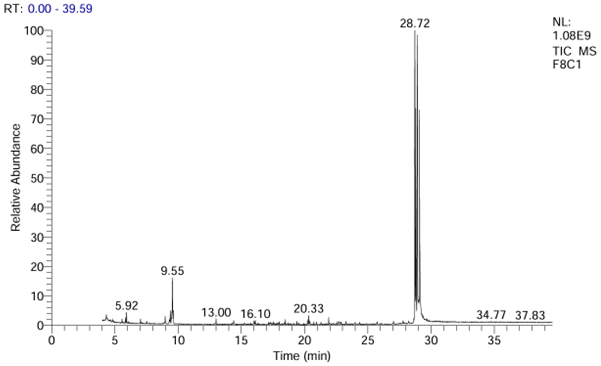 | |
| (**c**) | (**d**) | |
| 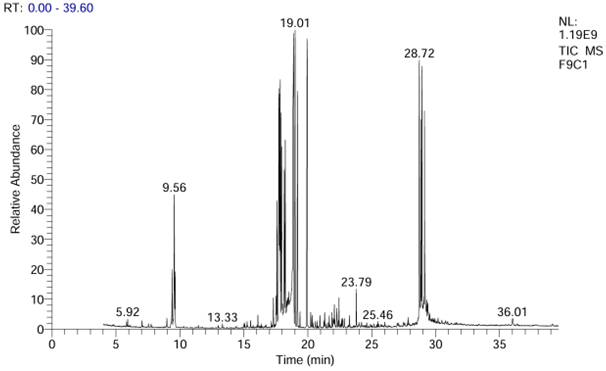 | | |
| (e) | | |

**Figure (2).** GC-MS chromatograms of fraction 5 (a), fraction 6 (b), fraction 7 (c), fraction 8 (d), and fraction 9 (e) of methanolic extract.

**Table (2): GC-MS results of the last fractions separated from methanolic extract**

|  | **t_R_** | **Compound name** | **Molecular formula** | **Molecular mass(g/mol)** | **Area%** | **SI** |
| --- | --- | --- | --- | --- | --- | --- |
| **Fraction 5** | 5.84 | Ethanamine, 2TMS | C_8_H_23_NSi_2_ | 189 | 0.78 | 708 |
|  | 5.93 | Lactic acid, 2TMS | C_9_H_22_O_3_Si_2_ | 234 | 1.83 | 953 |
|  | 8.98 | Benzoic Acid, TMS | C_10_H_14_O_2_Si | 194 | 3.39 | 932 |
|  | 9.38 | TMS, Quinoline | C_12_H_15_NSi | 201 | 1.40 | 845 |
|  | 9.56 | Phosphoric acid, 3TMS | C_9_H_27_O_4_PSi_3_ | 314 | 0.92 | 907 |
|  | 10.84 | Nonanoic acid, TMS | C_12_H_26_O_2_Si | 230 | 3.00 | 904 |
|  | 18.18 | Caffeine | C_8_H_10_N_4_O_2_ | 194 | 81.59 | 764 |
|  | 18.37 | Theobromine | C_7_H_8_N_4_O_2_ | 180 | 1.86 | 770 |
|  | 20.32 | Hexadecanoic acid, TMS | C_19_H_40_O_2_Si | 328 | 2.76 | 917 |
|  | 22.26 | trans-9-octadecenoic TMS ester | C_21_H_42_O_2_Si | 354 | 0.80 | 769 |
|  | 22.53 | Octadecanoic acid, TMS | C_21_H_44_O_2_Si | 356 | 0.65 | 884 |
|  | 26.08 | Monopalmitin TMS | C_25_H_54_O_4_Si_2_ | 474 | 1.02 | 847 |
| **Fraction 6** | 5.94 | Lactic acid, 2TMS | C_9_H_22_O_3_Si_2_ | 234 | 1.82 | 954 |
|  | 9.56 | Phosphoric acid, 3TMS | C_9_H_27_O_4_PSi_3_ | 314 | 14.26 | 855 |
|  | 11.21 | Erythrono-1,4-lactone, (E)-, 2TMS | C_10_H_22_O_4_Si_2_ | 262 | 1.34 | 891 |
|  | 14.25 | Butanoate, 4TMS- | C_16_H_40_O_5_Si_4_ | 424 | 4.95 | 897 |
|  | 16.01 | Octanedioic acid, 2TMS | C_14_H_30_O_4_Si_2_ | 318 | 34.19 | 688 |
|  | 16.13 | Xylose, 4-TMS | C_17_H_42_O_5_Si_4_ | 438 | 1.73 | 658 |
|  | 17.33 | Azelaic acid, 2TMS | C_15_H_32_O_4_Si_2_ | 332 | 20.29 | 765 |
|  | 19.15 | p-Coumaric acid, 2TMS | C_15_H_24_O_3_Si_2_ | 308 | 2.98 | 895 |
|  | 20.34 | Hexadecanoic acid, TMS ester | C_19_H_40_O_2_Si | 328 | 6.26 | 889 |
|  | 20.54 | 7-Methylxanthine, 2TMS | C_12_H_22_N_4_O_2_Si_2_ | 310 | 1.22 | 884 |
|  | 24.10 | 5-chloro-2'-desoxytris-O-Uridine TMS | C_18_H_35_ClN_2_O_5_Si_3_ | 478 | 3.67 | 801 |
|  | 25.47 | D-Galactose, 5TMS | C_21_H5_2_O_6_Si_5_ | 540 | 1.37 | 783 |
|  | 26.08 | Monopalmitin TMS | C_25_H_54_O_4_Si_2_ | 474 | 2.36 | 833 |
|  | 28.71 | Catechin TMS | C_30_H_54_O_6_Si_5_ | 650 | 1.94 | 693 |
|  | 28.91 | Epicatechin TMS | C_30_H_54_O_6_Si_5_ | 650 | 1.61 | 750 |
| **Fraction 7** | 5.57 | 3-Pyridinol, TMS | C_8_H_13_NOSi | 167 | 0.61 | 791 |
|  | 5.93 | Lactic acid, 2TMS | C_9_H_22_O_3_Si_2_ | 234 | 1.05 | 953 |
|  | 7.04 | Ethanolamine, 3TMS | C_11_H_31_NOSi_3_ | 277 | 0.82 | 680 |
|  | 7.53 | 3-Hydroxyisobutyric acid, 2TMS | C_10_H_24_O_3_Si_2_ | 248 | 1.09 | 771 |
|  | 8.98 | Benzoic Acid, TMS | C_10_H_14_O_2_Si | 194 | 1.41 | 866 |
|  | 9.39 | TMS-Quinoline | C_12_H_15_NSi | 201 | 4.10 | 78 |
|  | 9.55 | Glycerol, 3TMS | C_12_H_32_O_3_Si_3_ | 308 | 4.17 | 724 |
|  | 11.21 | Erythrono-1,4-lactone, (E)-, 2TMS | C_10_H_22_O_4_Si_2_ | 262 | 0.67 | 866 |
|  | 14.25 | Butanoate, 4TMS | C_16_H_40_O_5_Si_4_ | 424 | 2.06 | 888 |
|  | 15.98 | Octanedioic acid, bis-TMS | C_14_H_30_O_4_Si_2_ | 318 | 3.49 | 899 |
|  | 16.10 | Xylose, 4TMS | C_17_H_42_O_5_Si_4_ | 438 | 0.97 | 664 |
|  | 17.31 | Azelaic acid, 2TMS | C_15_H_32_O_4_Si_2_ | 332 | 1.33 | 801 |
|  | 17.52 | Methyl-α-D Fructofuranoside, 4TMS | C_19_H_46_O_6_Si_4_ | 482 | 1.01 | 682 |
|  | 18.71 | Indole-3-carboxaldehyde, TMS | C_12_H_15_NOSi | 217 | 0.88 | 893 |
|  | 18.85 | α-D-Glucopyranose, 5TMS | C_21_H_52_O_6_Si_5_ | 540 | 0.70 | 718 |
|  | 20.33 | Hexadecanoic acid, TMS | C_19_H_40_O_2_Si | 328 | 2.15 | 901 |
|  | 22.23 | Uridine, 3TMS | C_18_H_36_N_2_O_6_Si_3_ | 460 | 3.81 | 652 |
|  | 22.53 | Octadecanoic acid, TMS | C_21_H_44_O_2_Si | 356 | 0.86 | 872 |
|  | 25.46 | D-Galactose, 5TMS | C_21_H_52_O_6_Si_5_ | 540 | 0.97 | 783 |
|  | 28.72 | Catechin TMS | C_30_H_54_O_6_Si_5_ | 650 | 33.82 | 566 |
|  | 28.92 | Epicatechin TMS | C_30_H_54_O_6_Si_5_ | 650 | 33.21 | 819 |
|  | 31.60 | Scopelin-TMS | C_28_H_50_O_9_Si_4_ | 642 | 0.82 | 672 |
| **Fraction 8** | 5.83 | Ethanamine, 2TMS | C_8_H_23_NSi_2_ | 189 | 0.41 | 749 |
|  | 5.92 | Lactic acid, 2TMS | C_9_H_22_O_3_Si_2_ | 234 | 1.16 | 946 |
|  | 7.52 | 3-Hydroxyisobutyric acid, 2TMS | C_10_H_24_O_3_Si_2_ | 248 | 0.50 | 616 |
|  | 9.38 | TMS-Quinoline | C_12_H_15_NSi | 201 | 2.18 | 878 |
|  | 9.55 | Glycerol, 3TMS | C_12_H_32_O_3_Si_3_ | 308 | 8.72 | 875 |
|  | 16.10 | Xylose, 4TMS | C_17_H_42_O_5_Si_4_ | 438 | 0.76 | 687 |
|  | 17.52 | Methyl-α-D Fructofuranoside, 4TMS | C_19_H_46_O_6_Si_4_ | 482 | 1.83 | 673 |
|  | 20.33 | Hexadecanoic acid, TMS | C_19_H_40_O_2_Si | 328 | 1.07 | 893 |
|  | 20.41 | Yohimbine | C_21_H_26_N_2_O_3_ | 354 | 0.50 | 826 |
|  | 23.27 | α-DL-Arabinopyranose, 4TMS | C_17_H_42_O_5_Si_4_ | 438 | 1.92 | 675 |
|  | 28.72 | Catechin TMS | C_30_H_54_O_6_Si_5_ | 650 | 41.29 | 662 |
|  | 28.92 | Epicatechin TMS | C_30_H_54_O_6_Si_5_ | 650 | 39.65 | 656 |
| **Fraction 9** | 5.92 | Lactic acid, 2TMS | C_9_H_22_O_3_Si_2_ | 234 | 0.38 | 945 |
|  | 8.98 | Benzoic Acid, TMS | C_10_H_14_O_2_Si | 194 | 0.49 | 929 |
|  | 9.39 | TMS-Quinoline | C_12_H_15_NSi | 201 | 2.62 | 886 |
|  | 9.56 | Glycerol, 3TMS | C_12_H_32_O_3_Si_3_ | 308 | 6.36 | 878 |
|  | 13.33 | Erythritol (4TMS) | C_16_H_42_O_4_Si_4_ | 410 | 0.40 | 887 |
|  | 15.25 | L-Rhamnose, 4TMS | C_18_H_44_O_5_Si_4_ | 452 | 0.38 | 763 |
|  | 16.10 | Xylose, 4-TMS | C_17_H_42_O_5_Si_4_ | 438 | 0.74 | 680 |
|  | 17.31 | Methyl-α-D Fructofuranoside, 4TMS | C_19_H_46_O_6_Si_4_ | 482 | 1.21 | 693 |
|  | 17.95 | Fructose 5TMS | C_21_H_52_O_6_Si_5_ | 540 | 12.26 | 888 |
|  | 18.87 | Hexopyranose, 5TMS | C_21_H_52_O_6_Si_5_ | 540 | 33.0 | 876 |
|  | 19.94 | D-Glucopyranose, 5TMS | C_21_H_52_O_6_Si_5_ | 540 | 14.37 | 839 |
|  | 20.33 | Hexadecanoic acid, TMS | C_19_H_40_O_2_Si | 328 | 0.45 | 898 |
|  | 24.00 | Pimelic acid (2TMS) | C_13_H_28_O_4_Si_2_ | 304 | 0.49 | 582 |
|  | 24.20 | Mannonic acid, 1,4-lactone, TMS | C_18_H_42_O_6_Si_4_ | 466 | 0.45 | 685 |
|  | 28.72 | Catechin TMS | C_30_H_54_O_6_Si_5_ | 650 | 14.22 | 736 |
|  | 28.93 | Epicatechin TMS | C_30_H_54_O_6_Si_5_ | 650 | 12.17 | 712 |

**SI: match factor**

**t_R_: Retention time**
